# Brain Metastasis in Breast Cancer Patients—Need for Improvement

**DOI:** 10.3390/cancers12113190

**Published:** 2020-10-30

**Authors:** Isabell Witzel, Leticia Oliveira-Ferrer, Volkmar Müller

**Affiliations:** Department of Gynecology, University Medical Center Hamburg, 20246 Hamburg, Germany; ferrer@uke.de (L.O.-F.); vmueller@uke.de (V.M.)

**Keywords:** breast cancer, brain metastases, treatment, diagnosis, HER2, hormone receptor, triple negative

## 1. Background

The incidence of brain metastases (BM) in breast cancer patients is increasing. This is probably a result of improved survival rates with long-lasting remissions even in metastatic breast cancer patients. BM are a sign of advanced disease with currently limited treatment strategies and usually short survival times. Although BM are a rare event after initial diagnosis of early breast cancer with a 5 year incidence of 1.7% [1], BM occur in 30–50% of patients with metastatic disease [2,3], and 7.2% of patients have BM at first diagnosis of metastatic disease [4]. Risk factors that were identified at initial diagnosis for the development of subsequent BM were larger tumor size, node-positive disease, no pCR after neoadjuvant chemotherapy and HER2-positive or triple-negative subtype [5].

## 2. Open Questions and Future Directions of Clinical and Experimental Research

To improve treatment and possibly prevention of BM, better insights into mechanisms of brain tropism are required, and, therefore, research in this area is of high clinical relevance. The biology of BM is poorly understood. The ability to pass the blood–brain barrier and to survive and adapt within the new microenvironment may also determine the ability of tumor cells to metastasize to the brain in addition to a genetic predisposition of the tumor cell.

Treatment strategies for patients with BM are not well defined, as, until recently, these patients were excluded from many clinical trials. Therefore, evidence is based mostly on small cohort studies. As systemic treatments have limitations to be delivered through the blood–brain barrier, treatment of BM consists of neurosurgery and/or radiotherapy. Recently, various clinical trials have investigated the systemic treatment of BM in breast cancer patients with immunotherapeutic options and small molecules. With an increasing attention to the high clinical need for better evidence, some trials specifically allowed the inclusion of patients with active BM [6,7], and we hope to see more data from randomized trials in the future.

Finally, screening for BM, even in high-risk cohorts, has not been introduced into clinical routine. In addition, strategies to prevent patients from developing BM are unknown.

## 3. The Special Issue on Brain Metastases in Breast Cancer

In the context described above, it is the aim of this Special Issue to bundle current knowledge and new findings. This extends from basic research on the biology of BM in breast cancer to an overview about efforts to characterize features of breast cancer patients with BM in clinical cohorts and to updates about current and future treatment strategies. Therefore, this Special Issue should be of interest to basic researchers as well to clinicians involved in this emerging field.
